# Stress Analysis of Periodontal Tissue in en Masse Retraction With Integration of Maxillary Anterior Teeth: A Three-Dimensional Finite Element Method Study

**DOI:** 10.7759/cureus.68277

**Published:** 2024-08-31

**Authors:** Ran Saito, Hiroya Ozaki, Kenji Fushima, Hirofumi Arisaka

**Affiliations:** 1 Anesthesiology, Kanagawa Dental University, Yokosuka, JPN; 2 Orthodontics, Kanagawa Dental University, Yokosuka, JPN; 3 Dentistry and Orthodontics, Kanagawa Dental University, Yokosuka, JPN

**Keywords:** anchor screw, stress analysis, finite element method, maxillary anterior teeth, en masse retraction

## Abstract

Objective: To simulate the en masse traction technique with the integration (EMTI) of six maxillary anterior teeth using a finite element model (FEM) and explore various protocols for maxillary protrusion. The study aimed to investigate root displacement and stress distribution in the periodontal ligament (PDL) by varying the retraction position and direction of EMTI applied to the maxillary anterior teeth. No actual participants were involved.

Materials and methods: The FEM model included six teeth (central and lateral incisors and canines) with a PDL thickness of 0.3 mm. The model encompassing the alveolar bone (ALB) and EMTI had 180,528 elements and 47,836 nodes. The EMTI integrated six anterior teeth via a 0.9-mm-diameter stainless steel lingual wire, equipped with three moment arms extending toward the root apex: one midline (central arm) and two distal to the canines (lateral arms). The position and direction of the traction force applied to the three moment arms of the EMTI were varied to assess crown and apex displacement, as well as PDL stress.

Results: Lingual tipping was consistent across all protocols, emphasizing controlled incisor tipping. The application of horizontal traction at 10 mm and traction at 7 mm from the central and lateral arms of the EMTI, respectively, demonstrated the most uniform stress distribution across the PDL of all six anterior teeth.

Conclusions and clinical significance: The FEM analysis results suggest that the new EMTI method, which retracts the maxillary anterior teeth as a unit, is effective for tooth movement and PDL stress distribution. The EMTI technique, with its specific traction protocols and emphasis on controlled tipping, appears to be a promising approach for addressing maxillary protrusions.

## Introduction

The application of orthodontic force requires maximizing the speed of tooth movement while minimizing iatrogenic side effects. In cases of maxillary protrusion, a frequently used method is the extraction of the first premolar, followed by utilizing the resultant space to retract the maxillary anterior teeth towards the palate, aiming to correct their labial inclination. The palatal retraction of the maxillary anterior teeth using a multibracket appliance (MBA) risks causing uncontrolled tipping. Uncontrolled tipping is attributed to orthodontic force being applied to the teeth's crowns, which are far from the center of resistance [1]. From a biological perspective of tooth movement, external apical root resorption, a common iatrogenic issue during orthodontic treatment, is most frequently observed in the maxillary anterior teeth [2], with the extent of tooth movement being identified as a risk factor [3]. Consequently, uncontrolled tipping not only results in aesthetic irregularities in tooth alignment but also appears to contribute to the onset of apical root resorption.

One of the clinical challenges in orthodontics is to establish a treatment method that allows for controlled tipping of the maxillary incisors in cases of maxillary protrusion [4-14]. Several alternative methods have been reported for retracting the six maxillary anterior teeth as a unit using lingual appliances [10-14], as opposed to conventional MBA [4-9]. These methods are noted for their simpler mechanics, eliminating concerns about play and friction between brackets and wires, as well as potential side effects on adjacent teeth. When combined with skeletal anchorage devices positioned on the palate, these approaches are anticipated to be utilized effectively in the future [10-14]. In this study, we introduced a novel force system known as the en masse traction technique with integration (EMTI) for the maxillary anterior teeth. In EMTI, six maxillary anterior teeth are integrated using a stainless steel (SS) wire attached to the lingual side, and three moment arms extending from the lingual wire are pulled together as a unit by a palatal anchorage device. The EMTI is believed to offer superior axial control compared to conventional multibracket techniques when performing lingual traction of anterior teeth and can be expected to suppress apical stress concentration and subsequent external apical root resorption.

During orthodontic tooth movement, remodeling processes in periodontal tissues are initiated by alterations in the stress-strain distribution within the periodontal ligament (PDL) caused by intra-alveolar root displacement [15]. Pressure and tension within the PDL certainly cause various cellular responses, leading to periodontal tissue remodeling, and this process is called mechano-transduction [16,17]. This process involves osteoclast and osteoblast differentiation, both of which are derived from the blood. Histological studies have revealed a hyalinized avascular area in the PDL during orthodontic tooth movement, which causes a temporary pause in tooth movement [18]. Efficient tooth movement necessitates avoiding hyalinization and promoting periodontal tissue remodeling by suppressing excessive stress or strain that causes blood flow disturbances within the PDL.

Understanding stress distribution within the PDL is vital for designing orthodontic forces based on the biological principles of tooth movement. This study involved a three-dimensional finite element model (FEM) of an upper protrusion model, assuming six anterior teeth with labial inclination. We aimed to simulate the biomechanics of the EMTI technique by integrating six maxillary anterior teeth using FEM. Our goal was to explore different protocols for maxillary protrusion and investigate root displacement and stress distribution in the PDL by varying the retraction position and direction of the EMTI applied to the maxillary anterior teeth.

## Materials and methods

**Figure 1:**
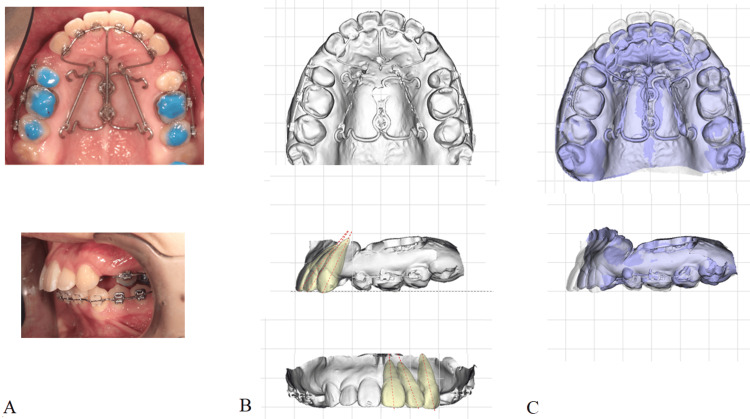
Clinical application on the maxillary anterior teeth using EMTI A: Intraoral photographs of a maxillary protrusion case. En-masse traction of six anterior teeth was performed by pulling the three arms of the EMTI toward the palatal anchorage device. B: Digital dental model (DM) at the onset of the EMTI application. Dotted lines in the frontal and sagittal views depict the tooth axes of each central, lateral, and canine tooth. C: The DM after seven months of EMTI traction was superimposed on the DM at the beginning. EMTI: En masse traction technique with the integration The images in this figure are original illustrations of clinical examples to explain the EMTI appliance.

**Figure 2:**
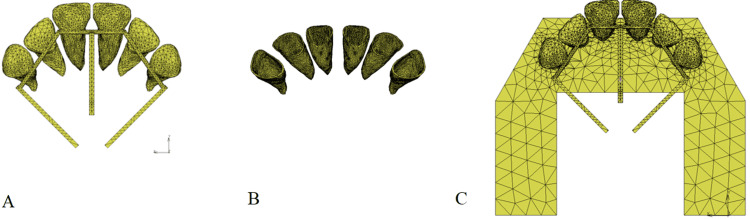
Finite element model (FEM) of maxillary anterior teeth with EMTI. A: Representation of six maxillary anterior teeth with EMTI; B: Depiction of the PDL of the six anterior teeth; C: Complete FEM model comprising teeth, EMTI, PDL, and ALB FEM: Finite element model, EMTI: En masse traction technique with the integration, PDL: Periodontal ligament, ALB: Alveolar bone The images in this figure are original illustrations.

In a clinical case of EMTI (Figure 1), en masse traction of the maxillary anterior teeth was performed by retracting the three moment arms toward the palatal anchorage device. Following the clinical example, the EMTI was reconstructed using the FEM model developed in this study (Figure 2).

The FEM model comprised six teeth in total: central incisors, lateral incisors, and canines on both sides of the maxilla, based on average Japanese anatomy for tooth dimensions (Table 1, Figure 2). Each tooth model was composed of isotropic tetrahedral elements. The axial angles of each tooth were set on the FEM model with reference to a typical upper protrusion to which EMTI was applied (Figure 1B). The axial angles of the central incisors, lateral incisors, and canines relative to the occlusal plane were set at 55.8°, 56.8°, and 67.8° in the sagittal view, and 86.9°, 72.4°, and 79.5° in the frontal view, respectively. The PDL, with a constant thickness of 0.3 mm, was constructed around each tooth, and an alveolar bone (ALB) was constructed. The EMTI, equipped with moment arms extending toward the root apex, integrated the six anterior teeth using a 0.9-mm-diameter stainless steel wire.

**Table 1:** Tooth dimensions of the FEM Tooth height: distance from the root apex to the incisal edge or canine cusp; Root length: distance from the alveolar crest to the root apex; Crown width: mesiodistal distance of the crown FEM: Finite element model

	Tooth height (mm)	Root length (mm)	Crown width (mm)
Central incisors	22.5	13.5	8.2
Lateral incisors	20.3	12.4	7.3
Canines	25.8	16.9	7.0

The FEM model, including the alveolar bone (ALB) and EMTI, comprised 180,528 elements and 47,836 nodes. The 3D solid models were created using Fu-sion360 (Autodesk Inc., San Francisco, CA, USA), and 3D finite element analysis was performed using Marc/Mentat (MSC Software Corp., Newport Beach, CA, USA). The Young's modulus and Poisson's ratio for the tooth, PDL, ALB, and wire were determined based on previous studies (Table 2) [8,19,20].

**Table 2:** Young's modulus and Poisson’s ratio of each element PDL: Periodontal ligament, ALB: Alveolar bone, EMTI: En masse traction technique with the integration

Elements	Young's modulus (Mpa)	Poisson's ratio
Tooth	20,000	0.3
PDL	0.05	0.3
ALB	2,000	0.3
EMTI	200,000	0.3

**Figure 3:**
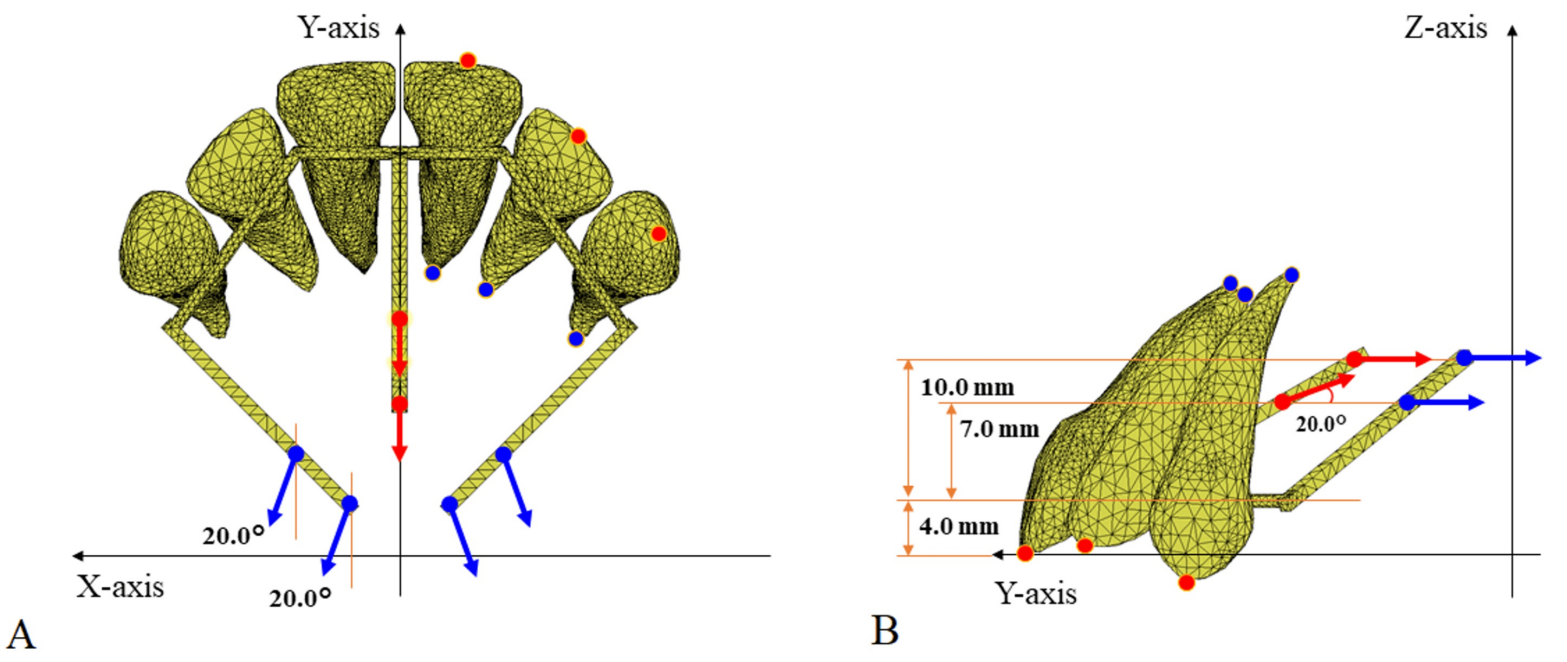
Coordinate system and traction conditions The XY(A) axis represents the occlusal planes, while the YZ(B) axis denotes the mid-sagittal plane. The red and blue arrows illustrate the traction points along with their respective heights and directions applied to the central and lateral arms. The red and blue circles represent the crown and apex of the tooth on the left side, respectively, and their displacements were analyzed in three dimensions. The images in this figure are original illustrations.

To analyze the displacement of each tooth in three dimensions, an occlusal plane coordinate system was established, with the XY plane representing the occlusal plane and the YZ plane representing the midsagittal plane (Figure 3). A lingual wire of EMTI integrated with the six anterior teeth was placed at a height of 4.0 mm perpendicular to the occlusal plane. The EMTI is equipped with three moment arms extending from the lingual wire toward the root apex: one in the midline between the central incisors (central arm), and two on the left and right distal to the canines (lateral arm).

The traction point and direction of the moment arms change, as shown in Figure 3. Traction points were set apically along the moment arm from the lingual wire attached to the anterior teeth in the Z coordinate at 7.0 mm and 10.0 mm for both the central and lateral arms. The central arm was pulled posteriorly along the midsagittal plane parallel to the Y-axis when the traction point was 10.0 mm and upward by 20° when the traction point was 7.0 mm. For the lateral arms, both the 7.0 mm and 10.0 mm traction points were pulled posteriorly parallel to the Y axis in the sagittal view (YZ plane) and 20° laterally in the occlusal view (XY plane).

Table 3 lists the six traction protocols. Concerning the traction of the central arm, three patterns emerged: posterior traction at a 20-degree angle upward from the 7 mm traction point, posterior traction horizontally from the 10 mm traction point, and no traction. Regarding traction of the lateral arms, two patterns of posterior traction in the sagittal plane were observed originating from the 7 mm and 10 mm traction points. In total, six traction patterns were analyzed.

**Table 3:** Traction protocols

	Central arm	Lateral arm
A	7 mm point traction with 20 degrees upward	10 mm point traction
B	10 mm point traction	10 mm point traction
C	No traction	10 mm point traction
D	7 mm point traction with 20 degrees upward	7 mm point traction
E	10 mm point traction	7 mm point traction
F	No traction	7 mm point traction

A traction force of 2N was applied from each traction point in the set traction direction, and a three-dimensional finite element analysis was performed. The displacement of the crown and root apex points on the left side and the stress distribution in the PDL were analyzed and compared for each protocol.

## Results

**Figure 4:**
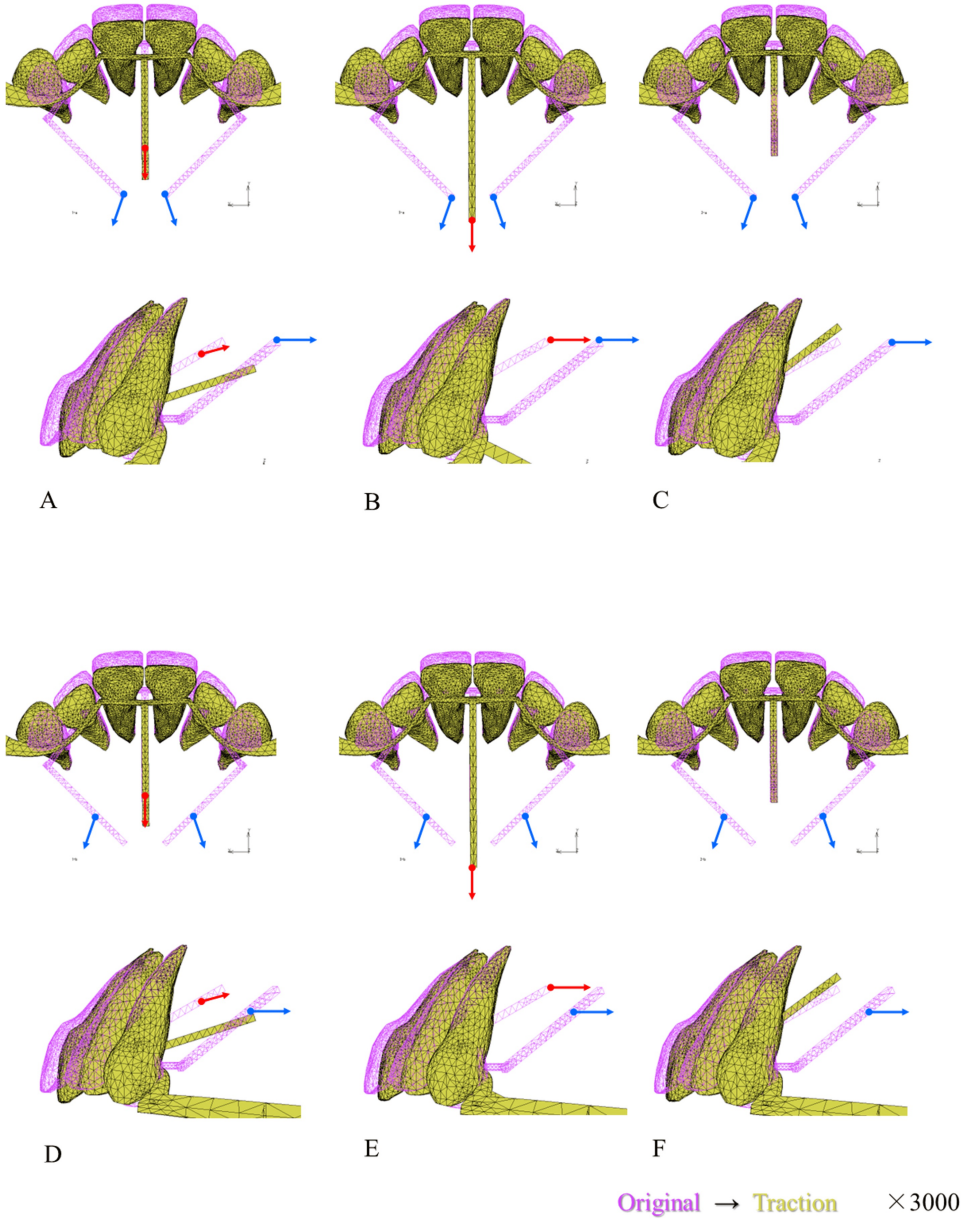
Displacement of maxillary anterior teeth in six protocols (visual representation) The six protocols (A to F) correspond to the definitions listed in Table 3. Displacements were demonstrated in the occlusal and sagittal views at 3000x magnification. The red and yellow wireframes illustrate the original and displaced positions by EMTI, respectively. Red lines indicate the traction points and directions applied to the central arm, whereas blue lines depict those applied to the lateral arms. EMTI: En masse traction technique with the integration The images in this figure are original illustrations.

Figure 4 shows the displacement patterns of the six protocols in the occlusal and sagittal views at 3000x magnification. From the sagittal view in all protocols, the lingual inclination of the central incisor was observed without obvious labial displacement of the root apex, indicating controlled tipping. From the occlusal view, when 10 mm traction was applied to the lateral arms (protocols A, B, and C from Table 3), the lateral displacement of the crown was noticeable, in contrast to when 7 mm traction was applied to the lateral arms (protocols D, E, and F from Table 3).

**Figure 5:**
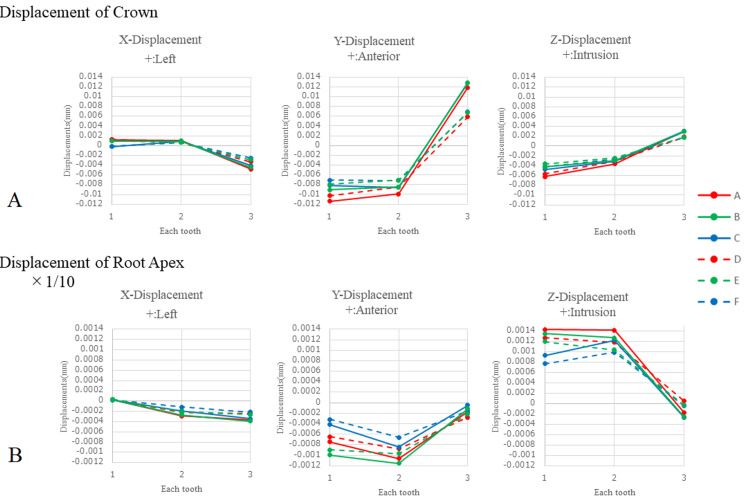
Displacement of crown and root apex points of maxillary anterior teeth in six protocols The displacement of the crown (A) and root apex (B) points of the maxillary anterior teeth was expressed along the X-, Y-, and Z-coordinates in the six protocols. The six protocols (A to F) correspond to the definitions listed in Table 3. The root apex point displacement was demonstrated at 10x magnification of the crown displacement. The numbers 1, 2, and 3 on the horizontal axis correspond to the central incisor, lateral incisor, and canine, respectively.

Figure 5 illustrates the initial displacement of the crown and root apex (10x magnification of the crown) of each tooth along the X-, Y-, and Z-directions of the coordinates for each traction protocol. In the y-coordinate system, the displacement of the crowns of the central and lateral incisors was consistently negative across all protocols, indicating lingual displacement. Despite the lingual inclination of the tooth axes of the central and lateral incisors (Figure 4), the root apex was not displaced labially but rather showed a slightly negative Y value, indicating lingual displacement (Figure 5). All traction protocols resulted in anterior displacement of the canine crown with a positive Y-displacement value, while a slight posterior displacement of the root apex was observed.

For the Z coordinate, the crowns of the central and lateral incisors showed negative values, indicating extrusive movement, whereas the root apex exhibited intrusive movement with positive values for all traction protocols. The intrusive movement of the root apex of the central incisor was smaller in protocols C and F, in which no traction was applied to the central arm than in the other protocols. In terms of the Z displacement, the crown of the canine displayed a slight intrusive displacement, whereas the root apex showed minimal displacement. On the X-coordinate, both the crown and root of the canine exhibited negative values, indicating lateral displacement.

**Figure 6:**
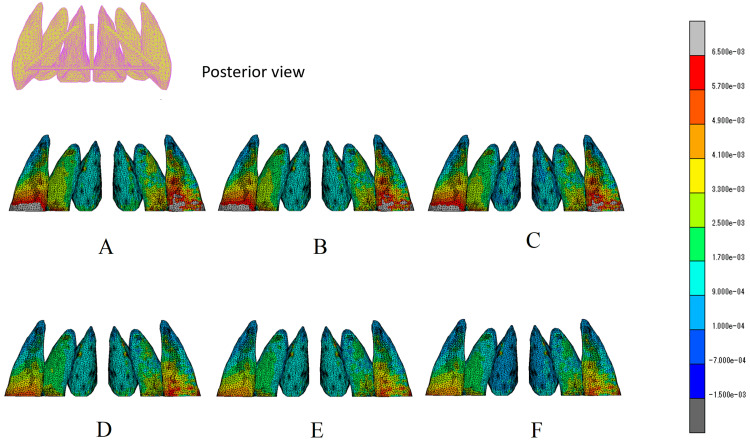
The von Mises stress distribution of PDL of maxillary anterior teeth in six protocols (visual representation) Stress levels in the PDL were visually represented in a posterior view and depicted using a color map. The six protocols (A to F) corresponded to the definitions listed in Table  3. PDL: Periodontal ligament The images in this figure are original illustrations.

The distribution of stress at the PDL-ALB interface is illustrated in the posterior view, with the color map depicting the von Mises stress located in the right corner (Figure 6). In cases where the central arm experienced no traction (protocols C and F), the stress distribution in the PDL of the central incisor was lower than that in the other traction protocols. In traction protocols A, B, and C, in which traction was applied 10 mm from the lateral arms, a high concentration of stress were observed around the cervical area of the canine. In protocols D and E, stress was more evenly distributed throughout the PDL of the six anterior teeth than in the other protocols.

**Figure 7:**
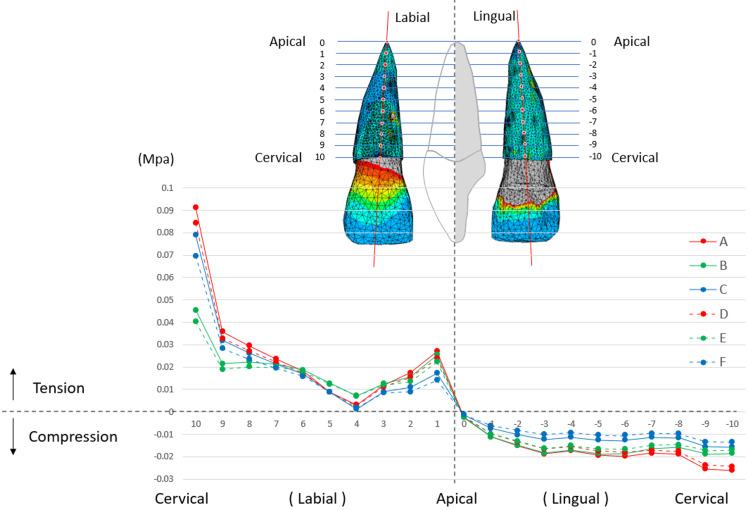
Stress in PDL along the labiolingual points of the central incisor Stress in the PDL was analyzed along the labiolingual points of the maxillary central incisor. The vertical axis indicates the von Mises stress in the PDL (MPa), with positive values representing tension stress and negative values representing compression stress. The horizontal axis indicates points ranging from the marginal point on the labial side (10) to the marginal point on the lingual side (-10), passing through the apical point of the root (0). The six protocols (A to F) correspond to the definitions listed in Table 3.

Stress at the PDL-ALB interface of the central incisor was assessed on both the labial and lingual sides. As depicted in Figure 7, the stress levels along the sagittal section were analyzed from the labial-cervical point through the root apex to the lingual-cervical point. The stress distribution between the labial and lingual sides is reversed at the root apex. Tensile stress was observed on the labial side, whereas compressive stress was evident along the entire length of the lingual side. Stress on the PDL of the central incisor may be influenced by variations in the traction protocols applied to the central arm. Specifically, within traction protocols A and D, where traction was executed at a 20-degree upward angle from a 7 mm traction point on the central arm, and protocols C and F, where no traction was applied to the central arm, the tensile stress at the labial-cervical point (10) was higher than that of protocols B and E, where traction was applied horizontally from a 10 mm traction point to the central arm. Overall, traction protocols B and E demonstrated a more even distribution of tensile stress on the labial side and compressive stress on the lingual side. In cases where no traction was applied to the central arm (C and F), the level of compressive stress was lower than that in the other protocols.

**Figure 8:**
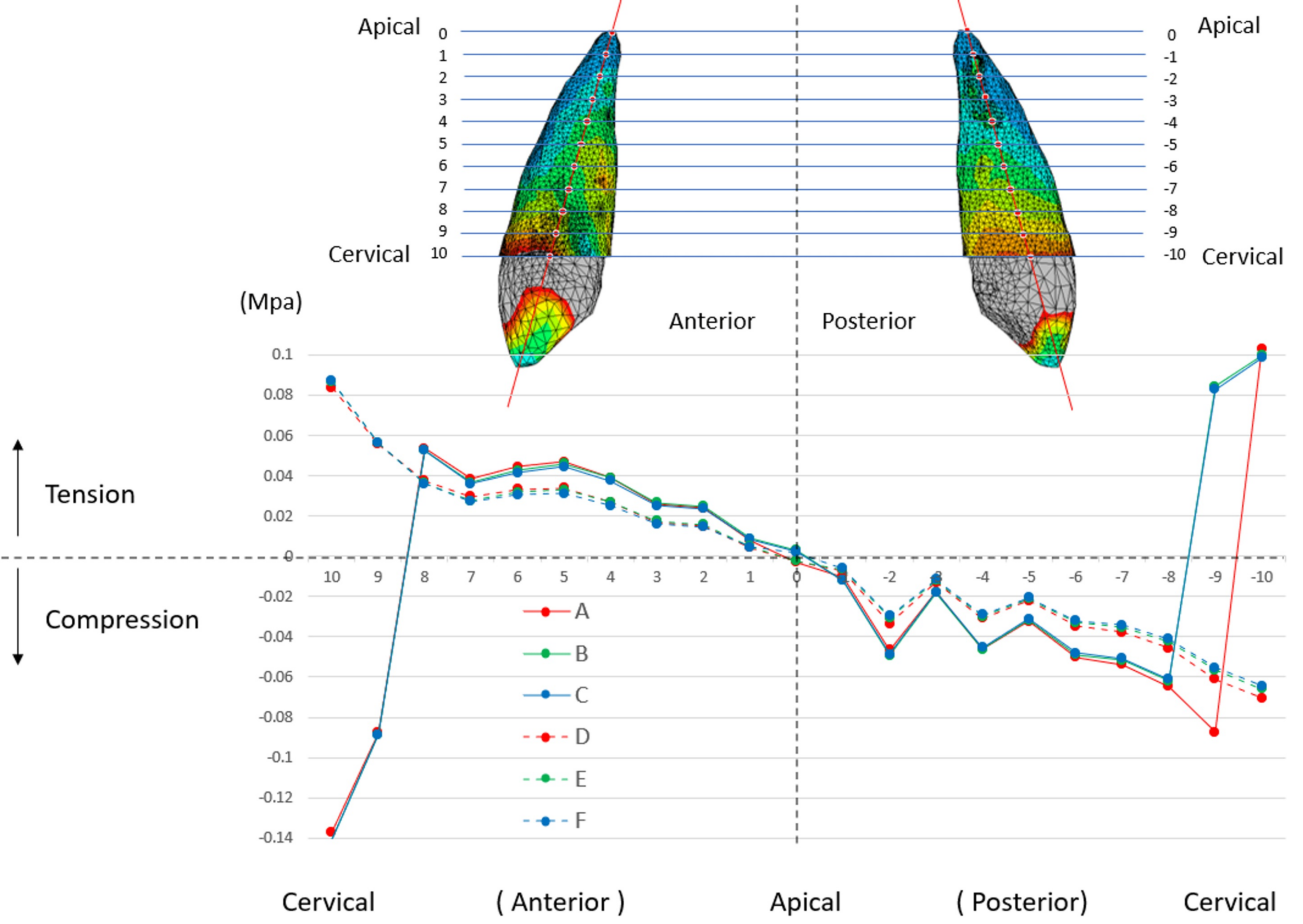
Stress in PDL along the anteroposterior points of canines The stress in PDL was analyzed along the anteroposterior points of the maxillary canine. The vertical axis indicates the von Mises stress in the PDL (MPa), with positive values representing tension stress and negative values representing compression stress. The horizontal axis indicates points ranging from the marginal point on the anterior side (10) to the marginal point on the posterior side (-10), passing through the apical point of the root (0). The six protocols (A to F) correspond to the definitions listed in Table 3. PDL: Periodontal ligament

Conversely, as illustrated in Figure 8, the stress on the canine PDL might be affected by differences in the traction protocols employed on the lateral arms. The stress levels along the sagittal section were analyzed from the anterior cervical point to the posterior cervical point through the root apex. In traction protocols D, E, and F, where traction was applied 7 mm from the lateral arms, the stress distribution between the anterior and posterior sides was reversed at the root apex. Tensile stress was observed on the anterior side, whereas compressive stress was evident along the entire length of the posterior side. In contrast, in traction protocols A, B, and C, in which traction was applied 10 mm from the lateral arms, significant compressive stress was observed at the anterior cervical points (10 and 9), whereas pronounced tensile stress was observed at the posterior cervical points (9 and -10). Tensile stress was observed from points 8 to 1, whereas compressive stress was observed from points -1 to -8. Within the range from 8 through 0 to -8, the stress distributions in traction protocols A, B, and C were similar but larger than those in traction protocols D, E, and F.

## Discussion

This study involved simulating the EMTI for six maxillary anterior teeth using the FEM and exploring various traction protocols, particularly in cases of maxillary protrusion. The investigation focused on six protocols that varied in the position and direction of traction on each arm, examining the displacement of the crown and root apex and stress distribution in the PDL.

In all the traction protocols, lingual tipping of the central and lateral incisors was observed in the sagittal view. The crown exhibited lingual displacement, whereas the root apex showed slight lingual displacement, a phenomenon known as controlled tipping of the incisors. Preventing uncontrolled tipping is crucial for the safe execution of orthodontic treatment [2,3].

In EMTI, the orthodontic force applied to the three arms generates a beneficial moment on the four front teeth. Several methods for directly bonding and pulling multiple teeth together without using brackets have been reported, along with their clinical effectiveness [10-14]. Reports have discussed adding long arms to the labial and palatal main arches of the MBA, exploring the effects of changes in traction direction, and using orthodontic anchor screws as a traction source [6-9]. In an MBA, due to the play at the bracket-wire interface, exerting a moment with long arms can be challenging, making it impossible to control each tooth uniformly [8]. Given that the EMTI integrates six anterior teeth into a group using a thick stainless-steel wire, the moment exerted by the three arms appears to act more directly than in the MBA, thereby inducing controlled tipping of the incisors.

In clinical situations, the EMTI is retracted to the skeletal anchorage device, as shown in Figure 1A. Using a palatal orthodontic implant, the anchorage point can be established deep in the palate [21], and the traction force and direction of EMTI can be effectively controlled to improve maxillary protrusion (Figure 1C).

The tooth movement required for orthodontic treatment must be safe and efficient. It is known that the stress generated in the PDL by orthodontic forces leads to the migration and differentiation of various cells, including osteoclasts and osteoblasts, which induce tooth movement [15]. The process through which external forces are translated into cellular responses is termed mechano-transduction and is believed to be triggered by the expression of appropriate stress while maintaining blood flow in the PDL [16,17]. Conversely, when the orthodontic force concentrates stress on a specific part of the PDL, thereby hindering blood flow, a hyalinized avascular area emerges, cell responses disappear, and tooth movement stagnates [18]. Hence, efficient tooth movement necessitates the following: (1) deviation of the tooth root in the alveolar fossa should not lead to stress concentration that could disrupt blood flow in a part of the PDL, and (2) appropriate stress should be consistently expressed throughout the PDL in the direction of tooth movement. To efficiently move the anterior teeth lingually using EMTI, achieving a more uniform stress development throughout the PDL of the six anterior teeth is desirable. In the color map (Figure 6), protocols D and E exhibited a more uniform stress distribution in the posterior view.

The canine crown was displaced anteriorly and laterally, whereas the root apex shifted slightly laterally in all traction protocols. The displacement was more pronounced when traction was applied to the lateral arms at a 10 mm point (traction protocols A, B, and C), correlating with the stress concentration at the cervical area of the canine (Figure 8). To avoid stress concentration, applying traction at the 7 mm point of the lateral arms is advisable (traction protocols D, E, and F). Examining the stress distribution within the PDL of the central incisor (Figure 7), the most uniform stress distribution was observed in protocols B and E, in which horizontal traction was applied from a 10 mm traction point to the central arm.

Based on the findings of this study, protocol E, which applied horizontal traction 10 mm from the central arm and traction 7 mm from the lateral arms, demonstrated the most uniform stress distribution across the PDL of all six anterior teeth, suggesting it as a clinically effective method. The location and direction of the traction forces applied to the three arms in the FEM model of protocol E closely resembled those of the EMTI used in clinical practice.

Although the results of this study support the validity of the clinical application of EMTI, it is important to acknowledge its limitations. The FEM reconstructed in this study was based on the standard tooth morphology of Japanese individuals. Future research should focus on validating the FEM simulation by replicating and analyzing the morphology of individual teeth and comparing it with real clinical outcomes. The FEM analysis conducted in this study was a linear analysis. It is suggested that further investigations utilizing nonlinear analysis methods [22] would be beneficial for a more comprehensive understanding in future studies.

## Conclusions

This study simulated the EMTI for six maxillary anterior teeth using the FEM. The position and direction of the traction force applied to the three moment arms of the EMTI were varied to assess crown and apex displacement, as well as PDL stress. Furthermore, this investigation revealed that lingual tipping of the central and lateral incisors was consistently observed in all traction protocols, with controlled tipping of the incisors being a notable phenomenon.

This study emphasizes the importance of achieving uniform stress distribution throughout the periodontal ligament of all six anterior teeth to ensure safe and efficient tooth movement. The application of horizontal traction 10 mm from the central arm and traction 7 mm from the lateral arms of the EMTI demonstrated the most uniform stress distribution across the PDL of all six anterior teeth, thereby establishing it as a clinically effective method.
